# The flavor and nutritional characteristic of four strawberry varieties cultured in soilless system

**DOI:** 10.1002/fsn3.346

**Published:** 2016-03-10

**Authors:** Li Liu, Mei‐Ling Ji, Min Chen, Ming‐yue Sun, Xi‐ling Fu, Ling Li, Dong‐Sheng Gao, Cui‐Ying Zhu

**Affiliations:** ^1^ State Key Laboratory of Crop BiologyCollege of Horticulture Science and EngineeringShandong Agricultural UniversityDaizong Road No. 61Tai'an 271018ShandongChina; ^2^ Shandong Collaborative Innovation Center for Fruit and Vegetable Production with High Quality and EfficiencyTaianChina

**Keywords:** Flavor, GC–MS, PCA, strawberry, volatile compounds

## Abstract

Strawberry fruits (cv. Benihoppe, Tochiotome, Sachinoka, and Guimeiren) were harvested and evaluated the flavor and nutritional parameters. By principal component analysis and hierarchical clustering analysis, differences were observed based on the volatile compounds composition, sugar and acid concentration, sweetness, and total soluble sugars/total organic acids of the four varieties. A total of 37, 48, 65, and 74 volatile compounds were identified and determined in cv. Benihoppe, Tochiotome, Sachinoka, and Guimeiren strawberry fruits extracted by head‐space solid‐phase microextraction (HS‐SPME), respectively. Esters significantly dominated the chemical composition of the four varieties. Furaneol was detected in cultivars of Sachinoka and Guimeiren, but mesifuran was only found in cv. Tochiotome. Tochiotome and Sachinoka showed higher content of linalool and (E)‐nerolidol. Sachinoka showed the highest content of total sugars and total acids. Guimeiren showed higher sweetness index than the other three cultivars. Firmness of Tochiotome was highest among all the varieties. The highest total soluble solids TSS value was found in cv. Sachinoka, followed by the Guimeiren and Tochiotome varieties. Sachinoka had the highest titratable acidity TA value. The content of ascorbic acid (AsA) of cv. Tochiotome was higher than the others, but there was no significant difference in cultivars of Benihoppe, Tochiotome, and Sachinoka. Fructose and glucose were the major sugars in all cultivars. Citric acid was the major organic acid in cv. Tochiotome, cv. Sachinoka, and cv. Guimeiren. Tochiotome had higher ratios of TSS/TA and total sugars/total organic acids than others, arising from its lower acid content. The order of the comprehensive evaluation score was Sachinoka>Guimeiren>Tochiotome>Benihoppe.

## Introduction

The strawberry (*Fragaria x ananassa* Duch.), one of the most appreciated cultivated fruits in the world, is popular for its pleasant flavors and nutritional qualities (Bourne 2002). It is not only consumed as fresh fruit but also an important food composition, such as ice cream, yogurt, and jam. Flavor, the most subtle and subjective of quality marker, is a consequence of the biosynthesis of a series of phytochemicals, including sugars, acids, and volatile compounds (Causse et al. 2001; Kader 2008; Klee 2010). The aroma of cultivated strawberries is an important attribute that greatly influences acceptability to the consumer. There are more than 360 volatile compounds that have been identified in strawberries consisting of esters, ketones, terpenes, furanones, aldehydes, alcohols, and sulfur‐containing compounds (Latrasse 1991). The typical strawberry aroma is provided by the compounds furaneol, *γ*‐decalactone, *γ*‐dodecalactone, methyl butanoate, ethyl butanoate, methyl hexanoate, ethyl hexanoate, linalool, and (E)‐nerolidol (Pérez et al. 2002; Azodanlou et al. 2003; Aharoni et al. 2004; Prat et al. 2014). The strawberry flavor is determined largely by the balance between the acids and sugars present (Kallio et al. 2000). Strawberry breeding has mainly focused on improving yields, disease resistance, fruit appearance, firmness, size, and storage properties. It is more challenging to manipulate the flavor quality factors than the others owing to numerous compounds participating in flavor formation. It is necessary to devote more attention to better assess and quantify the components of the flavor to improve this trait.

During the ripening stage, the metabolism of sugars, acids, and volatile compounds determines the fruit quality and appeal to the consumer. The nature and the concentration of sugars, acids, and aroma play important roles in organoleptic properties. What's more, these chemical components have impact on market quality of food industry. Therefore, much emphasis should be placed on sugars, acids, and volatile compounds in the breeding program.

In recent years, the commercial *Fragaria x ananassa* strawberry cultivars, such as Tochiotome, Sachinoka, Benihoppe, and Guimeiren, have been grown in greenhouses under soilless systems in China. Greenhouse production of strawberries may have some advantages, such as reduction in chemicals, increased yield per unit area, early production with high market prices, and better fruit quality. Prat et al. (2014) and Akhatou and Recamales (2014) have reported some improvement in nutritional and organoleptic qualities of strawberries in soilless growing systems. However, there are no reports of headspace solid‐phase microextraction–gas chromatography–mass spectrometry (HS–SPME–GC–MS) of volatile compounds in strawberries grown in soilless systems. The varieties above were chosen for this study for their commercial interest and economic importance in the area. Berries from these cultivars have excellent organoleptic properties with strong aroma. To the best of our knowledge, this study is also the first to determine the volatile composition in Benihoppe, Tochiotome, Sachinoka, and Guimeiren cultured in soilless system. The aim of this study was first to study the flavor and nutritional quality of the strawberries from each of these varieties and second to provide information on flavor compounds available for use in breeding programs.

## Materials and Methods

### Plant material and growth conditions

Strawberry fruit (*Fragaria x ananassa* Duch., octoploid cultivars) plants (*n *=* *200, each variety) were harvested using the same experimental and professional plantations located in an automated polycarbonate‐covered greenhouse of a horticultural science experimental station of Shandong Agriculture University, located in Tai'an, China (117°06′E, 36°15′N). The strawberry samples used for the analysis of the fruit quality consisted of four varieties, namely Benihoppe, Tochiotome, Sachinoka, and Guimeiren, cultivated in a soilless system using elevated horizontal troughs filled with organic substrate and irrigated with Hoagland's nutrient solution (as shown in Table S1).

In soilless growing systems, the radiation source in the greenhouse is provided by natural daylight. The temperature ranged from 25°C to 28°C during the day and 5°C to 10°C overnight. Relative humidity was maintained at 55–65%. The composition and concentration of the Hoagland's nutrient solution were shown in Table S1. At full maturity, at least 180 fruits (each replacement consisting of 60 fruits) from 180 plants for each cultivar were collected to generate a representative pooled fruit sample. Strawberries were packed into polystyrene punnets (capacity 500 g) and shipped to the laboratory (about 15 min). Some fruits of each sample were sliced and then homogenized in a blender, for testing volatile compounds and ascorbic acid (AsA) immediately. The remainders of the samples were stored at −4°C (about 3 days) for analysis of physicochemical parameters, sugars, and organic acids.

### Headspace solid‐phase microextraction analysis

A 2‐cm 50/30 *μ*m DVB/Carboxen/PDMS StableFlex fiber (Supelco, Inc., Bellefonte, PA) was used for the extraction of strawberry volatiles. Volatile compounds were extracted as described by Prat et al. (2014). At least 50 g of the purée of each sample with sodium chloride (3 g) was placed in a 50‐mL conical flask covered by an aluminum foil seal. For each treatment, three similar flasks were prepared for replications. The sample was equilibrated at 40°C on a magnetic stirring hotplate Heidolph 3001 (Sigma Aldrich, St. Louis, MO) for 30 min and extracted under stirring for 30 min at the same temperature. After extraction, the fiber was desorbed at 230°C for 2.5 min in the injection port for gas chromatography (GC).

### Analysis of volatile compounds by gas chromatography–mass spectrometry (GC–MS)

Volatile compounds were analyzed as described by Xu et al. (2015) on a GC‐MS‐QP 2010 Series system, which was equipped with a 30 m × 0.32 mm × 0.25 *μ*m Rtx‐5MS capillary column (Shimadzu, Tokyo, Japan). The samples (2 *μ*L) were injected in the splitless mode using a constant helium flow of 2.20 mL per min. The column temperature was maintained at 40°C for 2 min and programmed to increase by 6°C per min to 120°C, then by 8°C per min to 180°C, and finally by 15°C per min to 250°C at which temperature it was maintained for 5 min. The transfer line temperature was 200°C, and the mass spectrometer was operated in the electron impact (EI) ionization mode with an electron energy of 70 eV. Three replications of each sample were made in all cases (*n *=* *3). According to the previous report, 2‐octanol was as the internal standard (Xu et al. 2015). The quantification method of volatile compounds was calculated by the formula: *C* (*μ*g/L) = *A*_*c*_/*A*_*is *_× *C*_*is*_ (*μ*g/L), where C is the relative concentration of analyte; *C*_*is*_ is the final concentration of internal standard in the sample; *A*_*c*_ is the peak area of analyte; and *A*_is_ is the peak area of internal standard (Xu et al. 2015).

### Firmness, TA, TSS, and pH assessment

Firmness was determined using a Texture Analyzer TA‐XT‐Plus (Sterling, VA) equipped with a 2‐mm diameter flat probe. The maximum force of nine fruits for each variety developed during the test was recorded. Results are expressed in kilogram (kg).

Total acidity was determined by means of an acid‐base titration method, using 0.1 mol L^−1^ NaOH to pH 8.1. Results are expressed as grams of citric acid per 1 kg of fresh weight. Fruit‐soluble solid content was determined using an aliquot of strawberry juice at room temperature using a digital refractometer (Automatic Refractometer SMART‐1; Atago, Tokyo, Japan) and expressed as a percentage (Brix).

### HPLC analysis for sugars and organic acids

Sugars and organic acids were extracted as described by Xi et al. (2014). Approximately 1 g of homogenate was accurately weighed then diluted to 10.0 mL with ultrapure water (Millipore, Bedford, MA) and incubated for 20 min in a 35°C water bath. The supernatant was taken after centrifuging the samples at 10380g for 10 min (BHG‐Hermle Z 365, Wehingen, Germany). This extraction procedure was repeated three times, and the supernatants were combined. The liquid supernatant was filtered through a 0.22‐*μ*m, 13‐mm diameter syringe filter (Shanghaixingya Purification Material Factory, China). The filtered solution was then used for sugar and organic acid analysis.

Organic acid analyses were carried using a Waters series 515 chromatography unit (HPLC; Waters, Milford, MA) equipped with two 515 pumps and a 2487 dual UV detector (Waters Alliance 2695 HPLC) at a wavelength of 210 nm. The chromatographic separation of organic acids was carried out using NH_4_H_2_PO_4_ (10 mmol/L, pH 2.3) and methanol (98:2, v/v) as the mobile phase, with a flow rate of 0.8 mL per min, and samples were injected onto a Thermo Hypersil GOLD aQ (4.6 mm × 250 mm) column. The injection volume was 10 *μ*L.

Chromatographic separation of sugars was carried out using acetonitrile: water (75:25, v/v) as the mobile phase with a flow rate of 0.8 mL per min and a 5.0 *μ*m NH2 (4.6 mm × 250 mm) column (GL Sciences Inc., Torrance). The samples were injected onto a YMC‐Pack Polyamine II (4.6 mm × 250 mm) column. The injection volume was 10 *μ*L. Eluted peaks were detected with an RID‐10A refractive index detector (Shimadzu).

Soluble sugars and organic acids were quantified according to standard curves of authentic compounds. Extracts from three triplicate tissue samples were analyzed.

### HPLC analysis of AsA

Ascorbic acid analyses were performed on a high‐performance liquid chromatography system (HPLC; Waters, Milford, MA), equipped with two 515 pumps and a 2487 dual UV detector (Waters Alliance 2695 HPLC) at a wavelength of 260 nm. The chromatographic separation of AsA was carried out using NH_4_H_2_PO_4_ (10 mmol/L, pH 2.3) and methanol (98:2, v/v) as the mobile phase at a rate of 0.8 mL per min, and samples were injected onto a Thermo Hypersil GOLD aQ (4.6 mm × 250 mm) column. The injection volume was 10 *μ*L. Extracts from three triplicate tissue samples were analyzed. Results were expressed as grams of ascorbic acid per kilogram fresh weight (FW).

### Statistical analysis

All data were subjected to analysis of variance (ANOVA). The means were compared with least significant difference (LSD) test at a significance level of 95% (*P* = 0.05). SPSS Version 18.0 (SPSS Inc., Chicago, IL) was used for data analysis. Cluster analysis and heatmap visualizations of the centered data were performed using MultiExperiment Viewer (MeV) software (version 4.8.1; Dana‐Farber Cancer Institute, Boston, MA).

## Results and Discussion

### Identification of volatile compounds in four strawberry varieties

Fruit aroma of cultivated strawberries is an important attribute that greatly influences consumer acceptability. It is the result of a special assortment and relative quantities of a mixture of different metabolites (esters, terpenoids, aldehydes, alcohols, ketones, furans, lactones, and acids). The different proportions of the volatile components and the presence or absence of trace components often determine aroma properties. Therefore, the aim of our study was to investigate the volatile components of four varieties grown in a soilless system in a greenhouse.

All of the above‐mentioned families of volatile compounds have been reported in previous studies (Kaskas et al. 2005). In our study, 37, 48, 65, and 74 volatile compounds were identified in cv. Benihoppe, Tochiotome, Sachinoka, and Guimeiren strawberry fruits, respectively (Figure S1, Table S2).

**Figure 1:**
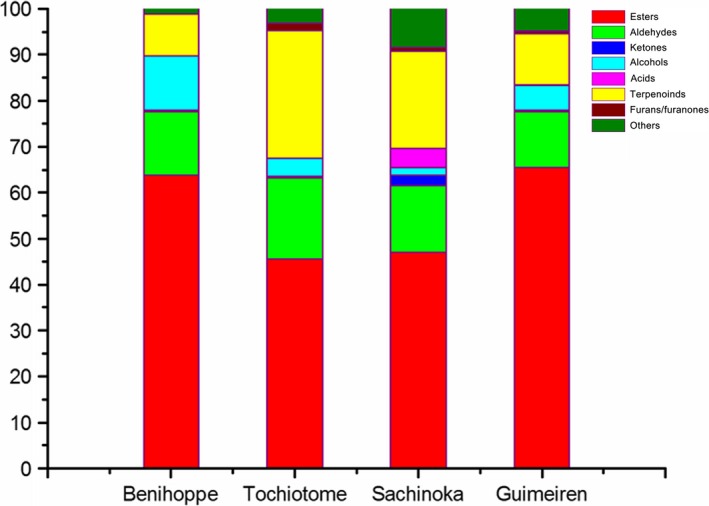
Percentage of volatile compounds found in the four strawberry varieties.

The major constituents by percentage in the headspace of all the four varieties were esters, aldehydes, and terpenoids (Fig.  1). In the samples of Benihoppe and Guimeiren, alcohols were the third and fourth most abundant group of compounds, respectively (Fig. 1). Ketones, acids, and furans/furanones were less abundant in the strawberry cultivars.

**Figure 2:**
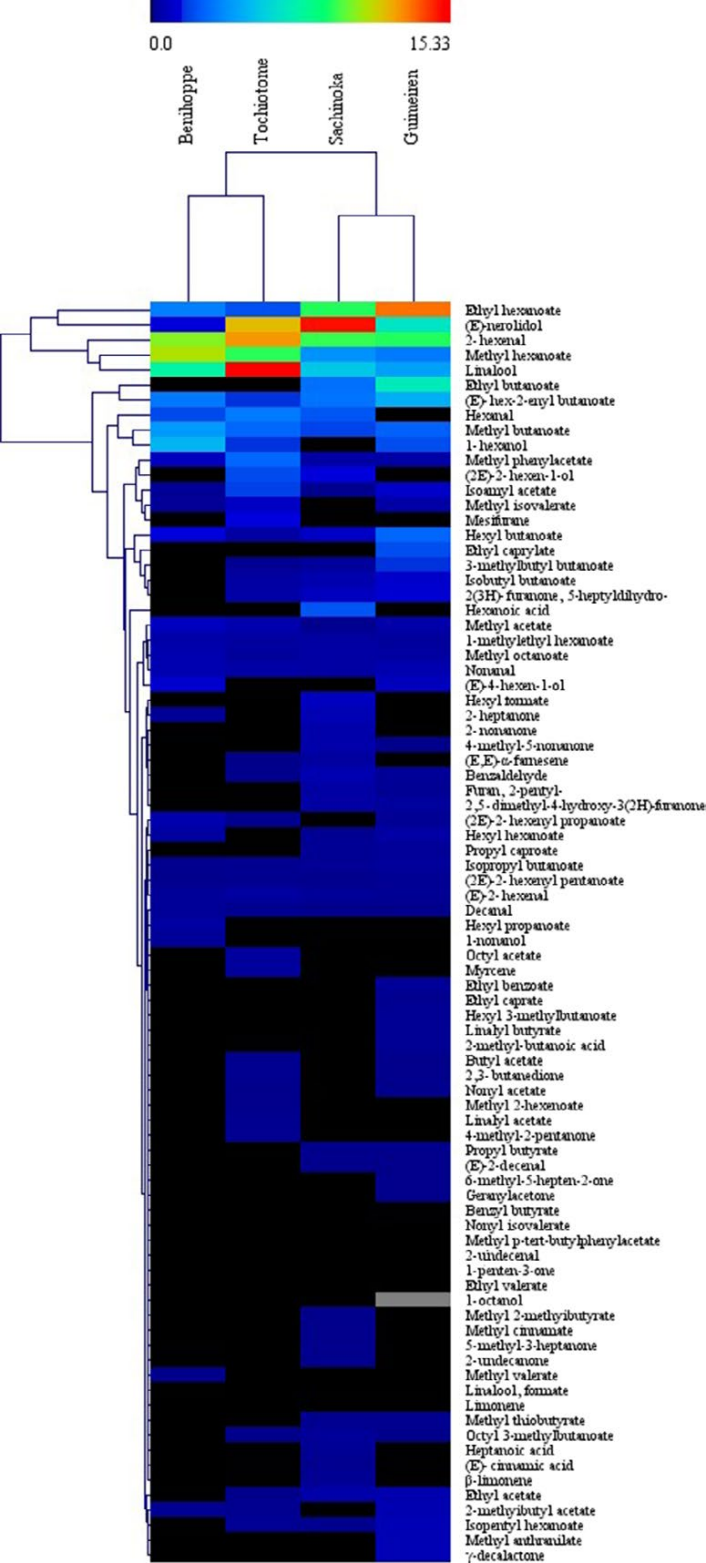
Hierarchical clustering and heat map visualization showing concentration of volatile aroma compounds identified in the strawberry samples. Dark and blue indicate low content. Green indicates intermediate content, and red indicates high content.

The diversity and concentration of volatile aroma compounds of the four strawberry varieties are visualized by using the hierarchical clustering and heatmap (Fig. 2). The clustering of aroma compounds shows that varieties of Benihoppe and Tochiotome are grouped closely, with high concentrations of the methyl butanoate, methyl hexanoate, 2‐hexenal, and linalool. Sachinoka and Guimeiren are grouped together and are specifically rich in ethyl hexanoate, (E)‐nerolidol, and ethyl butanoate.

According to the literature, esters are the most important group of strawberry aroma compounds responsible for fruity impressions (Latrasse 1991; Jetti et al. 2007). A previous study has shown that esters can account for 25–90% of the total volatile content in different cultivars of strawberry (Forney et al. 2000). In our case, esters significantly dominated the chemical compositions of the four varieties Benihoppe, Tochiotome, Sachinoka, and Guimeiren, accounting for 63.77%, 45.39%, 47.10%, and 65.47% of the total area quantified, respectively (Fig. 1). These results suggested that Benihoppe and Guimeiren had higher contents of esters than others, which implied that the two varieties might have a pleasant fruity aroma. Methyl butanoate, ethyl butanoate, methyl hexanoate, and ethyl hexanoate contribute the most to the fruity aroma of strawberries (Pérez et al. 2002; Azodanlou et al. 2003). Prat et al. (2014) reported that methyl butanoate gave sweet and fruity aroma, and ethyl butanoate was used as contributor to nutty notes, and the tropical fruit notes belong to ethyl hexanoate, according to the gas chromatography‐olfactometry (GC‐O) results. In our work, methyl butanoate, methyl hexanoate, and ethyl hexanoate were detected in all samples, while ethyl butanoate was found only in the Sachinoka and Guimeiren varieties (Fig. 2). In addition, the relative contents of the four characteristic esters accounted for 29–45% of the total esters, and Sachinoka and Guimeiren showed higher contents of the four esters, which suggested that they might have a more fruity and green aroma. Of the 67 esters identified among the four varieties, 16 ester compounds were common to all four genotypes. Each variety imparts some uniquely different kinds of esters (as shown in Table S3).

Only (E)‐2‐hexenal, nonanal, decanal, and 2‐hexenal were common to all samples (Table S2), especially 2‐hexenal, which ranging from 740.8 *μ*g/L to 1015.2 *μ*g/L, accounted for 65.72–76.53% of the aldehydes in all treatments. Tochiotome showed the highest levels of hexanal and (E)‐2‐hexenal, followed by Sachinoka, Benihoppe, and Guimeiren (Fig. 2). Hexanal gives asparagus and green impression, and (E)‐2‐hexenal gives sweet note, according to the GC‐O results (Prat et al. 2014). But (E)‐2‐hexenal is also linked to the fresh and green aroma. 1‐Hexanol showed higher relative concentration of 397.6 *μ*g/L in the Benihoppe variety, accounting for 86.84% of the total alcohol area quantified. In addition, 1‐hexanol has a fresh green odor reported by Jetti et al. (2007) and Prat et al. (2014). (2E)‐2‐hexen‐1‐ol was the only alcohol in cv. Sachinoka. Fatty acids represent precursors to aroma compounds. In this study, six acids were detected in the Sachinoka variety, but no acid was found in the case of Tochiotome. Branched‐chain acids 2‐methylbutanoic acid existed in the extracts of Benihoppe and Guimeiren.

Among the most important volatile compounds in the aroma of strawberries are 2, 5‐dimethyl‐4‐hydroxy‐3(2H)‐furanone (Furaneol) and its methoxy derivative (methoxyfuraneol, mesifuran) (Lavid et al. 2002). Furaneol and mesifuran are considered to be the two most important flavor‐active constituents of strawberry aroma, responsible for caramel and fruity notes (Prat et al. 2014). In our study, furaneol was detected in cultivars of Sachinoka and Guimeiren (Fig. 2), and there was significant difference in the relative content of this compound between the two varieties (Table S2). Mesifuran was only found in cv. Tochiotome. According to the literature, both furaneol and mesifuran were detected in some certain cultivars, but only one of the furans was found in some others as a result of cultivar differences, furaneol being less stable than mesifuran and other reasons (Douillard and Guichard 1989; Pérez and Sanz 2010).

The two most abundant terpenoid constituents were linalool and (E)‐nerolidol in the extracts of all varieties studied, accounting for 100%, 98.42%, 97.14%, and 100% of the terpenoid content in Benihoppe, Tochiotome, Sachinoka, and Guimeiren, respectively. A similar effect was reported by Samykanno et al. (2013) that linalool and (E)‐nerolidol dominated the terpenoid profile of two Australian‐grown strawberry varieties (Albion and Juliette). Myrcene and limonene were only found in Tochiotome. These two terpenoids were also reported in Albion and Juliette varieties (Samykanno et al. 2013 *)*. Also, (E, E)‐*α*‐farnesene has been reported as contributors to green‐fresh notes (Prat et al. 2014), which occurred in the varieties of Tochiotome and Sachinoka.

The principal component analysis is a multivariate statistical analysis method used to reduce the number of variables. PCA (Principal component analysis) was applied to identify which volatile compounds provided greatest ability to differentiate the varieties. The PCA result shows that the first three principal components have their cumulative reliability of 99.99% (Table 1). The first PC accounted for 43.28% of the total variance in the data set. The variety of Guimeiren was with the highest scores of the first principal component associated with ethyl acetate, ethyl butanoate, isopropyl butanoate, ethyl hexanoate, hexyl butanoate, and *γ*‐decalactone contributing to the first component. The variance ratio contribution of the second and third principal component was 34.17% and 22.54%, respectively (Table 1). The variables that contribute most to the second and third components are (E)‐2‐hexenal; (E)‐nerolidol; linalool; furan, 2‐pentyl‐; 2,5‐dimethyl‐4‐hydroxy‐3(2H)‐furanone; and mesifurane. We mainly carry out more lateral comparison for the volatile compounds selected, and they have a rank according to the scores of the three components to offer reference to strawberry breeding. The variety of Sachinoka got the highest scores of the second and third principal components (Table 2). Based on selection of volatile compounds indicators, principal component and factor analyses were used to calculate the comprehensive scores of volatile compounds. The comprehensive scores of each variety were in the order of Sachinoka>Guimeiren>Tochiotome>Benihoppe (Table 2). From the above analysis, the conclusion could be obtained that the variety of Sachinoka might have the most pleasant aroma of all the varieties.

**Table 1:** Principal component analysis for volatile compounds of strawberry

Component	Initial characteristic value		
	Eigenvalue	Variance (%)	Cumulative (%)
1	36.3587	0.4328	0.4328
2	28.7054	0.3417	0.7745
3	18.9359	0.2254	0.9999

**Table 2:** Principal components value of volatile compounds of strawberry

Variety	Component 1	Component 2	Component 3	Synthetic component
Benihoppe	−3.4017	−3.8673	−0.7111	−2.9539
Tochiotome	−5.7432	−2.3538	5.8333	−1.9751
Sachinoka	−0.1345	2.4099	3.93086	1.6513
Guimeiren	3.8427	−1.3651	1.95257	1.6368

### Effect of the variety on quality characteristics

Table 3 shows the quality characteristics of the Benihoppe, Tochiotome, Sachinoka, and Guimeiren cultivars. These quality characteristics include fruit shape, fruit weight, firmness, total soluble solids (TSS), titratable acidity (TA), and the ratio TSS/TA, which can influence the consumer's purchase intention. Fruits of Benihoppe and Guimeiren are conical, while Tochiotome fruit is long and conical, and Sachinoka is short and conical. The FW of single fruit is significantly different among the four varieties. Benihoppe, mean fruit weight 35 g of single fruit, is the largest among the four varieties. The fruit weights of Guimeiren, Tochiotome, and Sachinoka are 25.17, 21.50, and 21.67 g, respectively. The FW of single fruit of Benihoppe and Guimeiren was significantly greater than that of Tochiotome and Sachinoka, and there was no significant difference between Tochiotome and Sachinoka. In addition to weight, firmness is a desirable characteristic for consumers. Tochiotome shows higher firmness than others, which may suggest that it has good storage capacity and is suitable for supermarket sales.

**Table 3:** Physicochemical parameters determined in strawberry fruits of different varieties

Variety	Fruit shape	Fruit weight (g)	Firmness (kg)	TSS (Brix).	TA (g citric acid/kg)	TSS/TA
Benihoppe	Conical	35.00 ± 2.10a	0.03 ± 0.00b	10.27 ± 1.33b	5.63 ± 0.67b	18.26 ± 1.48ab
Tochiotome	Long conical	21.50 ± 2.74c	0.04 ± 0.00a	11.10 ± 0.90ab	5.43 ± 0.60b	20.65 ± 3.23a
Sachinoka	Short conical	21.67 ± 1.37c	0.02 ± 0.00bc	12.47 ± 0.50a	7.70 ± 0.72a	16.32 ± 2.18b
Guimeiren	Conical	25.17 ± 2.32b	0.02 ± 0.00c	11.13 ± 0.42ab	6.50 ± 0.82ab	17.25 ± 1.48ab

TA, titratable acidity.

TSS, total soluble solids.

Letters are comparisons of the four strawberry cultivars. Means with the same letter are not significantly different (*P *=* *0.05) by least significant difference (LSD) test.

Sugars and acids are most commonly associated with the taste of fruits, including strawberries, and are measured through TSS and TA. Statistical analysis showed that there were some significant differences of TSS and TA values between the four varieties. TSS, an indicator of the sugar content, is often expressed in terms of Brix. The highest TSS value was found in cv. Sachinoka, followed by the Guimeiren and Tochiotome varieties. Galletta et al. (1995) reported that the TSS value in strawberry fruit is generally in the range 7–12%, depending on the variety. In our study, the values for TSS ranged from 10.27% to 12.47%, which was higher than the previous results of TSS values for the Elsanta variety of 6.23% and 10–11% for the Camarosa variety grown under protected cultures (Paranjpe et al. 2003; Voc´a et al. 2007). Akhatou and Recamales (2014) measured lower levels of TSS in six cultivars of strawberries grown in a soilless system in Huelva (southwest Spain), ranging from 3.45% to 4.49%. Samykanno et al. (2013) reported TSS values of two Australian‐grown strawberry varieties—the Albion variety had levels of 10.8% and the Juliette variety had levels of 10.6%.

TA is expressed as a calculation of citric acid in this study. Sachinoka had the highest TA value, which was determined to have a slightly higher acid content than Guimeiren. The TA values of Benihoppe and Tochiotome were lower than the other two cultivars.

The TSS/TA ratio, often used as a measure of sweetness, is a good indicator of organoleptic evaluation for strawberries. Wozniak et al. (1997) reported that cultivars having TSS/TA of at least 7.00 are considered to have a sweet and acidic flavor. Ben‐Arie et al. (1984) noted that the ‘Wonderful’ accession that was perceived to have a sour‐sweet taste, varied from 11 to 16. In previous studies, TSS/TA values ranged from 5.0 to 13.0 for fruits of seven varieties (Akhatou and Recamales 2014; Ben‐Arie et al. 1984). It was noted that TSS/TA values for all the varieties studied here were much greater than 13.00, which may suggest that varieties grown in a soilless culture system might have a sweet and acidic flavor. In addition, Tochiotome had a higher TSS/TA than the others, as a result of its lower acid content (Table 4).

**Table 4:** Soluble sugars and ascorbic acid (AsA) content in strawberry fruits of different varieties expressed as g kg^−1^ of fresh fruit weight

Strawberry variety	Fructose	Glucose	Sucrose	Sorbitol	AsA (g kg^−1^)
Benihoppe	22.87 ± 0.95c	17.10 ± 1.11c	0.13 ± 0.01c	0.07 ± 0.00a	0.45 ± 0.19a
Tochiotome	24.60 ± 1.03c	21.37 ± 1.39b	4.86 ± 0.46b	0.03 ± 0.00b	0.46 ± 0.19a
Sachinoka	28.84 ± 1.20b	25.34 ± 1.65a	12.07 ± 1.15a	ND	0.44 ± 0.19a
Guimeiren	35.15 ± 1.46a	25.60 ± 1.67a	1.15 ± 0.11c	ND	0.39 ± 0.16b

Letters are comparisons of the four strawberry cultivars. Means with the same letter are not significantly different (*P *=* *0.05) by least significant difference (LSD) test.

ND, not detected.

Vitamin C plays an important role in the prevention of scurvy and maintenance of skin and blood vessels (Lee and Kader 2000). The content of AsA of cv. Tochiotome (5.39 g kg^−1^) was higher than the others, but there was no significant difference in cultivars of Benihoppe, Tochiotome, and Sachinoka (Table 4). The Vitamin C content in ripe strawberries was reported ranging from 0.19 g kg^−1^ to 1.1 g kg^−1^, for cultivars “Albion,” “Campineiro,” “Chandler,” “Camarosa,” and “Selva”(Pérez et al. 1997; Cordenunsi et al. 2005; Nunes et al. 2006; Ferreyra et al. 2007; Kafkas et al. 2007). In addition, Cardeñosa et al. (2015) reported that vitamin C content of cv. Primoris cultivated in soilless system was 0.20–0.25 mg g^−1^, which was much lower than our results. From the above mentioned, the four strawberry varieties in our study might be considered as rich in Vitamin C.

### Soluble sugar content

With regard to the soluble sugars, fructose, glucose, sucrose, and sorbitol were studied in strawberry fruits. As shown in Table 4, the contents of glucose, fructose, sucrose, and sorbitol were found to be significantly different (*P *<* *0.05) among some varieties. Fructose and glucose were the major sugars in all cultivars, accounting for >80% of total sugars. The levels of sugars varied between 22.87 (cv. Benihoppe) and 35.15 g kg^−1^ (cv. Guimeiren) for fructose and between 17.10 (cv. Benihoppe) and 25.60 g kg^−1^ (cv. Guimeiren) for glucose. Previous studies reported that the content of fructose was between 14.00 g kg^−1^ and 22.30 g kg^−1^ and the glucose content ranging from 11.45 to 20.70 g kg^−1^ for strawberries cultivated in a soil system (Akhatou et al. 2014; Bordonaba and Terry 2010). Compared with fructose and glucose, the fruit contained lower sucrose and sorbitol concentrations. Amount of sucrose was reported as about 15 g kg^−1^ for Albion variety at ripe stage grown on field in Chihuahua by Ornelas‐Paz et al. (2013), which was higher than our results. In our study, the highest content of sucrose was 12.07 g kg^−1^ for Sachinoka and the lowest content of sucrose was 0.13 g kg^−1^ for Benihoppe. What is more, sorbitol was not detected in the Sachinoka and Guimeiren varieties. The total amount of sugars was between 40.18 and 66.26 g kg^−1^, which is higher than reported by other authors on different cultivars (Akhatou and Recamales 2014).

### Organic acids

Organic acids are key components in strawberry flavor perception, as in most fruits (Medlicott and Thompson 1985). Of the organic acids measured by HPLC, citric acid, succinic acid, malic acid, and oxalic acid were relatively abundant, and trace amounts of lactic acid, acetic acid, and tartaric acid were also detected in some varieties (Table 5). Citric acid was the major organic acid in cv. Tochiotome, cv. Sachinoka, and cv. Guimeiren. Similarly, other researchers found that the major organic acid component was citric acid in strawberry cultivars Festival, Ventana, and Albion and other fruits (citrus, mango, medlar) (Yamaki 1989; Gil et al. 2000; Glewa et al. 2003; Basson et al. 2010; Ornelas‐Paz et al. 2013). The content of citric acid was highest in cv. Sachinoka (17.54 g kg^−1^). The citric acid values measured in our research ranged from 4.29 g kg^−1^ to 17.54 g kg^−1^, which was in keeping with those found in other studies (ranging from 4.40 g kg^−1^ to 15.00 g kg^−1^) (Sturm et al. 2003; Skupien and Oszmianski 2004). And similar contents of citric acid were reported in Albion (8.23 g kg^−1^), Korona (8.50 g kg^−1^), Camarosa (15.21 g kg^−1^), and Osmanli (18.85 g kg^−1^) (Kafkas et al. 2007; Keutgen and Pawelzik 2007; Ornelas‐Paz et al. 2013). Benihoppe had the largest amount of succinic acid (6.65 g kg^−1^) within its organic acid composition. The content of succinic acid was higher than in the range reported of 0.50–1.41 g kg^−1^, but a similar value was found for malic acid (0.98–1.89 g kg^−1^) (Akhatou and Recamales 2014). The content of malic acid in the tested four varieties was between 0.50 and 1.70 g kg^−1^, lower than the reported (2.00–2.80 g kg^−1^) for cultivars' Aromas, Diamante, Selva, Korona, Elsanta, Albion, and Osmanli (Pelayo et al. 2003; Kafkas et al. 2007; Keutgen and Pawelzik 2007; Ornelas‐Paz et al. 2013). Sturm et al. (2003) found that tartaric acid was detected in some varieties, in agreement with our results. The difference in content of total organic acids was obvious among the four varieties. The highest value was measured in the variety of Sachinoka, which was a combination of high total sugars and acids. It is noted that high sugars and acids were taken into account for screen breeding material. Compared with Sachinoka, the other three varieties had significantly lower content of total organic acids, and Tochiotome showed the lowest concentration of total organic acids.

**Table 5:** Organic acids content in strawberry fruits of different varieties expressed as g kg^−1^ of fresh fruit weight

Strawberry variety	Citric acid	Succinic acid	Malic acid	Oxalic acid	Lactic acid	Acetic acid	Tartaric acid
Benihoppe	5.20 ± 0.45c	6.65 ± 0.93a	0.50 ± 0.03d	1.67 ± 0.07a	0.54 ± 0.04b	0.54 ± 0.04a	0.55 ± 0.04a
Tochiotome	4.29 ± 0.37c	1.43 ± 0.20c	1.70 ± 0.10a	1.37 ± 0.06b	0.61 ± 0.05a	0.44 ± 0.05b	ND
Sachinoka	17.54 ± 1.52a	1.71 ± 0.24c	1.61 ± 0.10b	1.31 ± 0.06d	ND	ND	ND
Guimeiren	6.87 ± 0.60b	2.88 ± 0.40b	0.95 ± 0.05c	1.34 ± 0.06c	0.58 ± 0.04a	0.40 ± 0.05b	0.15 ± 0.01b

Letters are comparisons of the four strawberry cultivars. Means with the same letter are not significantly.

Different (*P *=* *0.05) by least significant difference (LSD) test.

ND, not detected.

Values of total sugars/total organic acids and the sweetness index are useful tools to characterize acceptance of fruit by consumers (Glewa et al. 2003; Basson et al. 2010; Bordonaba and Terry 2010; Veberic et al. 2010). There was significant difference in total sugars/total organic acids among the varieties (Table 6). The ratios of total sugars/total organic acids were in the order of Tochiotome>Guimeiren>Sachinoka>Benihoppe. Sachinoka and Guimeiren showed higher sweetness index than the other two varieties (Table 6). As shown in Figure S2, there was high correlation coefficients between sweetness index and total sugars (*r* = 0.985).

**Table 6:** Total sugars and total acids content, and the ratio of total sugar/total acids in strawberry fruits of different varieties in a soilless growing system

Strawberry variety	Total sugars	Total acids	Total sugars/total acids	Sweetness index
Benihoppe	40.18 ± 2.03d	15.31 ± 1.71b	2.65 ± 0.41d	53.02 ± 2.46c
Tochiotome	50.86 ± 2.22c	9.94 ± 0.93c	5.15 ± 0.65a	63.96 ± 2.66b
Sachinoka	66.26 ± 2.52a	21.77 ± 2.23a	3.07 ± 0.37c	81.55 ± 3.06a
Guimeiren	61.90 ± 3.04b	13.02 ± 1.32b	4.79 ± 0.62b	81.86 ± 3.72a

Letters are comparisons of the four strawberry cultivars. Means with the same letter are not significantly different (*P *=* *0.05) by least significant difference (LSD) test.

ND, not detected.

**Figure 3:**
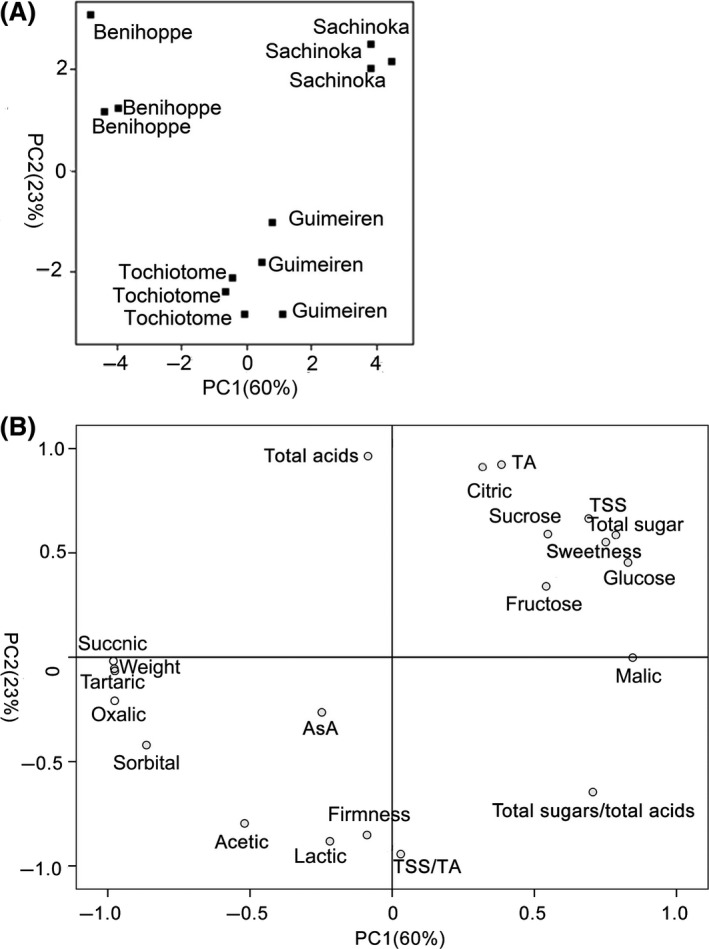
Scores (A) and loadings (B) of principal component analysis for the strawberry samples. In Figure 3B, “sweetness” refers to “sweetness index.”

PCA was used to control all steps of the visualization to generate more effective visual representations. The processing data of each variety were repetitive and all the varieties were well‐separated (Fig. 3A). Results of principal component analysis showed that the two principal components explained 83% of the total variance. The variance ratio contribution of the first principal component is about 60%. Total sugars, sweetness, total sugars/total acids, TSS, fructose, glucose, and sucrose are the chief contributor of the first component and play important role in fruit flavor (Fig. 3B). PC2 is positively correlated with the content of TA, citric acid, and total acids. As a result, the first principal component is regarded as the measure of sweetness, and also the second principal component is considered as the measure of sourness. The cultivar of Sachinoka in the positive parts of PC1 and PC2 implied higher sweetness and sourness. The variety of Benihoppe was located in the opposite part of PC1 and thus showed lower sweetness. Similarly, Tochiotome was associated with moderate sweetness and lower sourness. In this article, regarding relative variance contribution of each principal component as weight, the comprehensive evaluation score of each variety was calculated based o n the evaluation function of strawberry quality. The higher the comprehensive evaluation score is, the better the comprehensive quality will be. The overall quality of cv. Sachinoka was better than the others, as shown in Table S4.

## Conclusions

The comparison of different cultivars of strawberries grown in soilless systems revealed differences in nutritional and organoleptic qualities, mainly in flavor parameters. We identified 109 different volatile compounds in all cultivars. In our study, 37, 48, 65, and 74 volatile compounds were identified in cv. Benihoppe, Tochiotome, Sachinoka, and Guimeiren strawberry fruits, respectively. The clustering of aroma compounds shows that Benihoppe and Tochiotome are grouped closely and Sachinoka and Guimeiren are grouped together, and esters significantly dominated the chemical composition of the four varieties. Methyl butanoate, ethyl butanoate, methyl hexanoate, and ethyl hexanoate are considered to be important flavor‐active components. Sachinoka and Guimeiren showed higher content of these four typical esters. Furaneol was detected in cultivars of Sachinoka and Guimeiren, but mesifuran was only found in cv. Tochiotome. Linalool and (E)‐nerolidol were found to be the two major terpenoid constituents in the extracts of all varieties studied. Tochiotome and Sachinoka showed higher content of linalool and (E)‐nerolidol. The highest TSS and TA value was found in cv. Sachinoka. In addition, Sachinoka showed the highest content of total sugars and total acids. Tochiotome had a higher TSS/TA and total sugars/total organic acids than others, arising from its lower acid content. The content of AsA of cv. Tochiotome was higher than the others. Fructose and glucose were the major sugars in all cultivars. Citric acid was the major organic acid in cultivars of Tochiotome, Sachinoka, and Guimeiren. The results of PCA showed good discrimination among the four varieties, and the comprehensive evaluation score of Sachinoka was the highest. The results obtained in this work will be a useful reference for future strawberry‐breeding programs.

## Conflict of Interest

The authors declare that they have no competing interests.

## Supporting information


**Figure S1.** Venn diagram of volatile compounds among the four strawberry varieties.

**Figure S2.** Correlation coefficient (*r*) between sweetness index and total sugars of strawberry.

**Table S1.** The composition of Hogland's nutrient solution.

**Table S2.** Identification of compounds by gas chromatography–mass spectrometry (GC–MS) in Benihoppe, Tochiotome, Sachinoka and Guimeiren varieties.

**Table S3.** Esters special of each variety in a soilless growing system.

**Table S4.** Principal components value of quality markers of strawberry.
