# Supplementary figures and images for: The flavor and nutritional characteristic of four strawberry varieties cultured in soilless system
Source: Food Sci Nutr. 2016 Mar 10;4(6):858–68. doi: 10.1002/fsn3.346 (PMC5090650; doi:10.1002/fsn3.346)

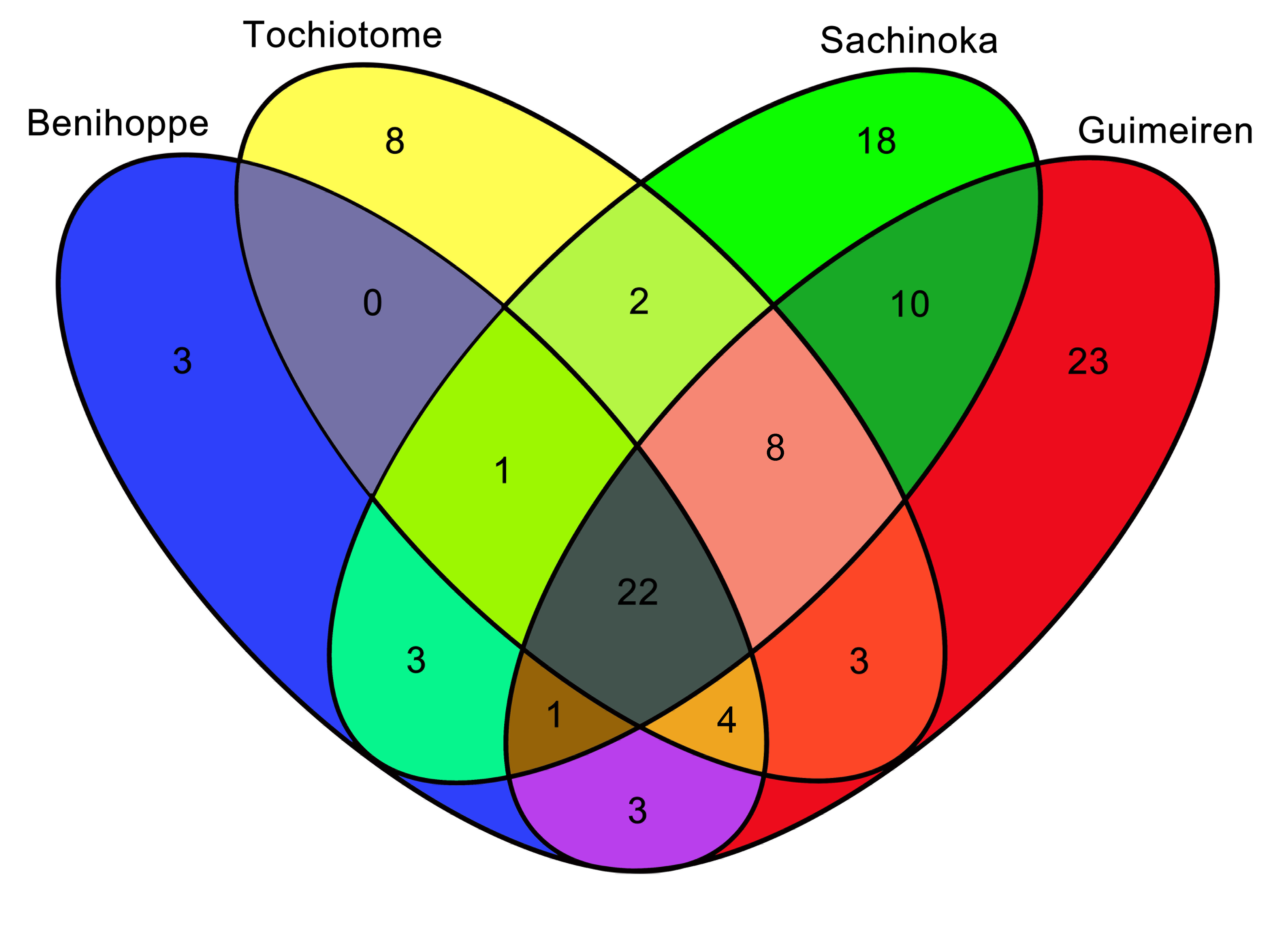

Supplement: Supplementary file 1 — Figure S1. Venn diagram of volatile compounds among the four strawberry varieties. [file FSN3-4-858-s001.tif]

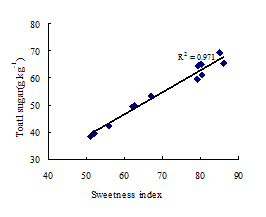

Supplement: Supplementary file 2 — Figure S2. Correlation coefficient (r) between sweetness index and total sugars of strawberry. [file FSN3-4-858-s002.tif]
