# Supplementary material for: The flavor and nutritional characteristic of four strawberry varieties cultured in soilless system
Source: Food Sci Nutr. 2016 Mar 10;4(6):858–68. doi: 10.1002/fsn3.346 (PMC5090650; doi:10.1002/fsn3.346)
Supplement: Supplementary file 3 — Table S1. The composition of Hogland's nutrient solution. [file FSN3-4-858-s003.doc]

**Table S1-The composition of Hogland’s nutrient solution**

| Names | Manufacturer | Concenration（mol/L） |
| --- | --- | --- |
| Ca(NO_3_)_2_ | Guoyao,Shanghai,China | 5×10^－3^ |
| KNO_3_ |  | 5×10^－3^ |
| KH_2_PO_4_ |  | 2×10^－3^ |
| MgSO_4_ |  | 4×10^－3^ |
| EDTA-Fe |  | 1×10^－4^ |
| H_3_BO_4_ |  | 3.7×10^－5^ |
| MnCl_2_·4H_2_O |  | 9.1×10^－6^ |
| CuSO_4_·5H_2_O |  | 3.2×10^－7^ |
| ZnSO_4_ .7H_2_O |  | 7.6×10^－7^ |
| H_2_MoO_4_·H_2_O |  | 5×10^－7^ |
