# Supplementary material for: The flavor and nutritional characteristic of four strawberry varieties cultured in soilless system
Source: Food Sci Nutr. 2016 Mar 10;4(6):858–68. doi: 10.1002/fsn3.346 (PMC5090650; doi:10.1002/fsn3.346)
Supplement: Supplementary file 4 — Table S2. Identification of compounds by gas chromatography–mass spectrometry (GC–MS) in Benihoppe, Tochiotome, Sachinoka and Guimeiren varieties. [file FSN3-4-858-s004.doc]

**Table S2-Identification of compounds by gas chromatography-mass spectrometry (GC–MS) in Benihoppe, Tochiotome, Sachinokaand Guimeiren varieties**

|  | CAS | | | Formula | | | Concentrations(µg/L) of compounds of strawberry variety | | | | | | | | | | |
| --- | --- | --- | --- | --- | --- | --- | --- | --- | --- | --- | --- | --- | --- | --- | --- | --- | --- |
|  |  |  |  |  |  |  | Benihoppe | | Tochiotome | | | | Sachinoka | | | | Guimeiren |
| **Esters** | | | | | | | | | | | | | | | | | |
| Methyl acetate | | 79-20-9 | C3H6O2 | | | 80.80±3.17a | | | | | 52.00±7.88b | | | 11.20±1.83c | | 48.00±5.89b | |
| Ethyl acetate | | 141-78-6 | C4H8O2 | | | ND | | | | | 6.40±0.50b | | | 44.80±4.92a | | 48.80±5.56a | |
| Methyl butanoate | | 623-42-7 | C5H10O2 | | | 328.0±18.47a | | | | | 226.4±16.70ab | | | 159.2±12.08b | | 220.8±14.00ab | |
| Methyl 2-methylbutyrate | | 868-57-5 | C6H12O2 | | | ND | | | | | ND | | | 7.20±1.73 | | ND | |
| Ethyl butanoate | | 105-54-4 | C6H12O2 | | | ND | | | | | ND | | | 248.0±27.70b | | 568.8±23.08a | |
| Butyl acetate | | 123-86-4 | C6H12O2 | | | ND | | | | | 12.0±1.74a | | | ND | | 5.60±0.60b | |
| Methyl valerate | | 624-24-8 | C6H12O2 | | | 6.40±0.49 | | | | | ND | | | ND | | ND | |
| Isopropyl butanoate | | 638-11-9 | C7H14O2 | | | 14.40±2..50bc | | | | | 12.80±0.20c | | | 17.60±1.83b | | 28.80±2.16a | |
| Isoamyl acetate | | 123-92-2 | C7H14O2 | | | 22.40±0.69c | | | | | 157.6±8.72a | | | 10.40±1.71d | | 92.00±2.12b | |
| 2-Methylbutyl acetate | | 624-41-9 | C7H14O2 | | | 19.20±0.61b | | | | | 14.40±1.31b | | | ND | | 56.00±8.63a | |
| Methyl thiobutyrate | | 2432-51-1 | C5H10OS | | | ND | | | | | ND | | | 12.80±0.69a | | 10.40±0.35a | |
| Propyl butyrate | | 105-66-8 | C7H14O2 | | | ND | | | | | ND | | | 5.60±0.50a | | 5.60±0.21a | |
| Ethyl valerate | | 539-82-2 | C7H14O2 | | | ND | | | | | ND | | | ND | | 4.80±0.49 | |
| Methyl hexanoate | | 106-70-7 | C7H14O2 | | | 888.0±17.36a | | | | | 748.8±31.61b | | | 314.4±22.89c | | 268.0±13.88d | |
| Ethyl hexanoate | | 123-66-0 | C8H16O2 | | | 277.6±0.12.70c | | | | | 184.8±12.08d | | | 742.4±20.39b | | 1071.2±22.53a | |
| **Table S2.** Continued | | | | | | | | | | | | | | | | | |
| 1-Methylethyl hexanoate | 2311-46-8 | | | C9H18O2 | | | 48.0±5.11a | | 29.6±1.44b | | | | 29.6±1.06b | | | | 25.6±2.20b |
| 3-Methylbutyl butanoate | 106-27-4 | | | C9H18O2 | | | ND | | 19.2±2.16b | | | | 24.8±2.12b | | | | 132.0±14.22a |
| Hexyl propanoate | 2445-76-3 | | | C9H18O2 | | | 29.60±0.72a | | ND | | | | 4.00±0.21b | | | | ND |
| (2E)-2-Hexenyl propanoate | 53398-80-4 | | | C9H16O2 | | | 49.6±3.49a | | 13.6±1.22b | | | | ND | | | | 18.4±1.38b |
| Methyl octanoate | 111-11-5 | | | C9H18O2 | | | 51.2±2.23a | | 25.6±2.88d | | | | 37.6±4.23b | | | | 32.8±1.40c |
| Methyl phenylacetate | 101-41-7 | | | C9H10O2 | | | 72.8±2.02b | | 228.0±6.97a | | | | 37.6±1.06c | | | | 36.8±4.45c |
| Hexyl butanoate | 2639-63-6 | | | C10H20O2 | | | 128.0±9.70b | | 36.0±1.39d | | | | 82.4±4.45c | | | | 226.4±26.44a |
| (E)-Hex-2-enyl butanoate | 53398-83-7 | | | C10H18O2 | | | 264.0±7.27b | | 132.0±4.26c | | | | 251.2±2.44b | | | | 382.4±14.76a |
| Octyl acetate | 112-14-1 | | | C10H20O2 | | | ND | | 26.4±2.10 | | | | ND | | | | ND |
| Isopentyl hexanoate | 2198-61-0 | | | C11H22O2 | | | ND | | 16.8±0.64b | | | | 14.4±0.72b | | | | 61.6±1.95a |
| nonyl acetate | 143-13-5 | | | C11H22O2 | | | ND | | 8.0±0.07a | | | | ND | | | | 5.6±0.21b |
| Hexyl hexanoate | 6378-65-0 | | | C12H24O2 | | | 30.4±0.72b | | 4.0±0.03d | | | | 16.8±0.93c | | | | 36.0±2.00a |
| Methyl isovalerate | 556-24-1 | | | C6H12O2 | | | 24.0±0.71c | | 85.6±0.99a | | | | ND | | | | 40.0±1.54b |
| Hexyl 3-methylbutanoate | 10032-13-0 | | | C11H22O2 | | | ND | | ND | | | | ND | | | | 17.6±0.42 |
| Benzyl butyrate | 103-37-7 | | | C11H14O2 | | | ND | | ND | | | | ND | | | | 3.2±0.21 |
| Octyl 3-methylbutanoate | 7786-58-5 | | | C13H26O2 | | | ND | | 7.2±0.08b | | | | 20.0±0.74a | | | | 6.4±0.14b |
| Isobutyl butanoate | 539-90-2 | | | C8H16O2 | | | ND | | 32.8±1.13c | | | | 51.2±2.08b | | | | 92.8±1.32a |
| Propyl caproate | 626-77-7 | | | C9H18O2 | | | ND | | ND | | | | 16.8±0.75b | | | | 23.2±1.39a |
| Ethyl caprylate | 106-32-1 | | | C10H20O2 | | | ND | | ND | | | | ND | | | | 182.4±14.36 |
| **Table S2.** Continued | | | | | | | | | | | | | | | | | |
| Ethyl caprate | 110-38-3 | | | C12H24O2 | | | ND | | ND | | | | ND | | | | 12.0±0.6 |
| Nonyl isovalerate | 7786-47-2 | | | C14H28O2 | | | ND | | ND | | | | ND | | | | 3.2±0.36 |
| Ethyl benzoate | 93-89-0 | | | C9H10O2 | | | ND | | ND | | | | ND | | | | 22.4±1.34 |
| Methyl anthranilate | 134-20-3 | | | C8H9NO2 | | | ND | | ND | | | | ND | | | | 60.0±6.22 |
| Methyl p-tert-butylphenylacetate | 3549-23-3 | | | C13H18O2 | | | ND | | ND | | | | ND | | | | 3.2±0.16 |
| Hexyl formate | 629-33-4 | | | C7H14O2 | | | ND | | ND | | | | 81.6±3.55 | | | | ND |
| Linalyl acetate | 115-95-7 | | | C12H20O2 | | | ND | | 11.2±2.95 | | | | ND | | | | ND |
| Methyl cinnamate | 103-26-4 | | | C10H10O2 | | | ND | | ND | | | | 6.4±0.68 | | | | ND |
| Linalyl butyrate | 78-36-4 | | | C14H24O2 | | | ND | | ND | | | | ND | | | | 15.2±1.39 |
| Linalool, formate | 115-99-1 | | | C11H18O2 | | | ND | | ND | | | | 4.0±0.17 | | | | ND |
| Hexyl acetate | 88230-35-7 | | | C8H16O2 | | | 1012.8±65.47a | | 452.0±18.02b | | | | 384.0±20.52b | | | | 292.0±21.55c |
| (Z)-hex-2-enyl acetate | 56922-75-9 | | | C8H14O2 | | | 1656.0±55.38a | | 1008.0±50.85b | | | | 467.2±18.71c | | | | 516.0±10.35c |
| (2E)-2-Hexenyl pentanoate | 35154-45-1 | | | C11H20O2 | | | 7.2±0.65c | | 14.4±2.54b | | | | 7.2±0.73c | | | | 18.4±1.49a |
| 4-Octenoic acid, methyl ester | 1732-00-9 | | | C9H16O2 | | | 13.6±1.13 | | ND | | | | ND | | | | ND |
| (E)-Hex-2-enyl hexanoate | 53398-86-0 | | | C12H22O2 | | | 65.6±6.35b | | 23.2±0.85d | | | | 40.8±3.18c | | | | 85.6±2.97a |
| Ethylene glycol di-n-butyrate | 105-72-6 | | | C10H18O4 | | | ND | | 35.2±2.36b | | | | 252.8±21.21a | | | | 267.2±13.44a |
| 2-Ethylhexyl butanoate | 25415-84-3 | | | C12H24O2 | | | 4.8±0.71ab | | ND | | | | 5.6±0.81a | | | | 4.0±0.52b |
| Butanoic acid, 1-methyloctyl ester | 69727-42-0 | | | C13H26O2 | | | ND | | ND | | | | ND | | | | 4.8±0.72 |
| Sec-octyl acetate | 54515-77-4 | | | C10H20O2 | | | 7.2±0.84b | | ND | | | | 20.8±2.32a | | | | ND |
| **Table S2.** Continued | | | | | | | | | | | | | | | | | |
| Heptan 2-yl butyrate | 39026-94-3 | | | C11H22O2 | | | ND | | ND | | | | 75.2±4.05a | | | | 14.4±1.39b |
| Methyl 2-hexenoate | 2396-77-2 | | | C7H12O2 | | | ND | | 8.0±0.67 | | | | ND | | | | ND |
| 2-Heptanol, acetate | 5921-82-4 | | | C9H18O2 | | | ND | | 7.2±0.92 | | | | ND | | | | ND |
| Octanoic acid, 3-hydroxy-,  methyl ester | 85549-54-8 | | | C9H18O3 | | | ND | | ND | | | | ND | | | | 6.4±0.50 |
| (Z)-pent-2-enyl butyrate | 42125-13-3 | | | C9H16O2 | | | ND | | ND | | | | ND | | | | 100.0±9.04 |
| Ethyl hex-2-enoate | 1552-67-6 | | | C8H14O2 | | | ND | | ND | | | | 2.4±0.49b | | | | 12.8±2.56a |
| Hexanethioic acid, S-propyl ester | 2432-78-2 | | | C9H18OS | | | ND | | ND | | | | ND | | | | 4.0±0.24 |
| 2-Ethylhexyl hexanoate | 16397-75-4 | | | C14H28O2 | | | ND | | ND | | | | ND | | | | 13.6±0.80 |
| 2-Methylbutyl hexanoate | 2601-13-0 | | | C11H22O2 | | | ND | | ND | | | | ND | | | | 4.0±0.58 |
| Propanoic acid, 2-methyl-,  nonyl ester | 10522-34-6 | | | C13H26O2 | | | ND | | ND | | | | ND | | | | 26.4±2.00 |
| 4-Hexen-1-ol, acetate | 72237-36-6 | | | C8H14O2 | | | ND | | ND | | | | 188.8±8.11 | | | | ND |
| 2-Heptadecanol, acetate | 56599-51-0 | | | C19H38O2 | | | ND | | ND | | | | 20.8±0.74 | | | | ND |
| Butanoic acid, 1-methyloctyl ester | 69727-42-0 | | | C13H26O2 | | | ND | | ND | | | | 40.8±1.39 | | | | ND |
| Hexanoic acid, 3-tridecyl ester |  | | | C19H38O2 | | |  | |  | | | | 9.6±0.35 | | | | ND |
| **Total concentrations of esters** |  | | |  | | | 5101.6 | | 3639.2 | | | | 3768.0 | | | | 5237.6 |
| **No. of esters** |  | | |  | | | 24 | | 31 | | | | 39 | | | | 51 |
| **Aldehydes** | | | | | | | | | | | | | | | | | |
| Hexanal | 66-25-1 | | | C6H12O | | | 168.8±7.41c | | 264.8±19.51a | | | | 196.8±11.40b | | | | ND |
| (E)-2-Hexenal | 6728-26-3 | | | C6H10O | | | 20.0±1.40b | | 30.4±2.27a | | | | 22.4±1.38b | | | | 19.2±3.61b |
| **Table S2.** Continued | | | | | | | | | | | | | | | | | |
| Nonanal | 124-19-6 | | | C9H18O | | | 54.4±3.21a | | 43.2±2.18b | | | | 34.4±4.43c | | | | 55.2±1.91a |
| Decanal | 112-31-2 | | | C10H20O | | | 24.0±1.39a | | 14.4±1.90b | | | | 8.8±1.23c | | | | 8.0±1.39c |
| 2-Hexenal | 505-57-7 | | | C6H10O | | | 837.6±64.63b | | 1015.2±56.95a | | | | 762.4±33.77c | | | | 740.8±30.67c |
| 2(3H)-Furanone, 5-heptyldihydro- | 104-67-6 | | | C11H20O2 | | | ND | | 41.6±5.49c | | | | 75.2±6.63ab | | | | 110.4±18.32a |
| (E)-2-Decenal | 3913-81-3 | | | C10H18O | | | ND | | ND | | | | 5.6±0.21a | | | | 9.6±0.58b |
| 2-Undecenal | 2463-77-6 | | | C11H20O | | | ND | | ND | | | | ND | | | | 3.2±0.28 |
| Benzaldehyde | 100-52-7 | | | C7H6O | | | ND | | 6.4±0.28c | | | | 54.4±2.80a | | | | 21.6±1.61b |
| **Total concentrations of aldehydes** |  | | |  | | | 1104.8 | | 1416.0 | | | | 1160.0 | | | | 968.0 |
| **No. of aldehydes** |  | | |  | | | 5 | | 7 | | | | 8 | | | | 8 |
| **Ketones** | | | | | | | | | | | | | | | | | |
| 6-Methyl-5-hepten-2-one | 110-93-0 | | | C8H14O | | | ND | | ND | | | | ND | | | | 7.2±0.64 |
| Geranylacetone | 3796-70-1 | | | C13H22O | | | ND | | ND | | | | ND | | | | 7.2±0.24 |
| 2,3-Butanedione | 431-03-8 | | | C4H6O2 | | | ND | | 13.6±0.88a | | | | ND | | | | 8.0±1.62b |
| 4-methyl-2-pentanone | 108-10-1 | | | C6H12O | | | ND | | 13.6±1.74 | | | | ND | | | | ND |
| 2-Heptanone | 110-43-0 | | | C7H14O | | | 22.4±2.83b | | ND | | | | 62.4±5.50a | | | | ND |
| 2-Nonanone | 821-55-6 | | | C9H18O | | | ND | | ND | | | | 44.8±6.43 | | | | ND |
| 4-Methyl-5-nonanone | 35900-26-6 | | | C10H20O | | | ND | | ND | | | | 48.0±4.74a | | | | 6.4±0.29b |
| 5-Methyl-3-heptanone | 541-85-5 | | | C8H16O | | | ND | | ND | | | | 9.6±1.41 | | | | ND |
| **Table S2.** Continued |  | | |  | | |  | |  | | | |  | | | |  |
| 1-Penten-3-one | 1629-58-9 | | | C5H8O | | | ND | | ND | | | | ND | | | | 3.2±0.58 |
| 2-Undecanone | 112-12-9 | | | C11H22O | | | ND | | ND | | | | 9.6±0.02 | | | | ND |
| **Total concentrations of ketones** |  | | |  | | | 22.4 | | 27.2 | | | | 174.4 | | | | 32.0 |
| **No. of ketones** |  | | |  | | | 1 | | 2 | | | | 5 | | | | 5 |
| **Alcohols** | | | | | | | | | | | | | | | | | |
| 1-Hexanol | 111-27-3 | | | C6H14O | | | 397.6±18.48a | | 133.6±11.18c | | | | ND | | | | 179.2±9.72b |
| (E)-4-Hexen-1-ol | 928-92-7 | | | C6H12O | | | 104.0±7.82a | | ND | | | | ND | | | | 76.8±10.99b |
| Cyclohexanol | 108-93-0 | | | C6H12O | | | 420.8±24.02a | | ND | | | | ND | | | | 164.8±9.12b |
| 1-Nonanol | 143-08-8 | | | C9H20O | | | 20.0±3.86 | | ND | | | | ND | | | | ND |
| 1-Octanol | 111-87-5 | | | C8H18O | | | ND | | ND | | | | ND | | | | 4.0±0.35 |
| (2E)-2-Hexen-1-ol | 928-95-0 | | | C6H12O | | | ND | | 177.6±14.55a | | | | 128.8±14.55b | | | | ND |
| **Total concentrations of alcohols** |  | | |  | | | 942.4 | | 311.2 | | | | 128.8 | | | | 424.8 |
| **No. of alcohols** |  | | |  | | | 4 | | 2 | | | | 1 | | | | 4 |
| **Acids** | | | | | | | | | | | | | | | | | |
| Hexanoic acid | 142-62-1 | | | C6H12O2 | | | ND | | ND | | | | 195.2±10.24 | | | | ND |
| 2-methyl-Butanoic acid | 116-53-0 | | | C5H10O2 | | | 1.6±0.24b | | ND | | | | ND | | | | 15.2±2.20a |
| Heptanoic acid | 111-14-8 | | | C7H14O2 | | | ND | | ND | | | | 19.2±1.23 | | | | ND |
| (E)-Cinnamic acid | 140-10-3 | | | C9H8O2 | | | ND | | ND | | | | 14.4±1.97 | | | | ND |
| Octanoic Acid | **124-07-2** | | | C8H16O2 | | | ND | | ND | | | | 70.4±6.49 | | | | ND |
| **Table S2.** Continued |  | | |  | | |  | |  | | | |  | | | |  |
| Myristic acid | | **544-63-8** | | | C14H28O2 | | | ND | | ND | | 4.0±0.29 | | | ND | | |
| Hexadecanoic acid | | 1957-10-3 | | | C16H32O2 | | | ND | | ND | | 43.2±2.17 | | | ND | | |
| **Total concentrations of acids** | |  | | |  | | | 1.6 | | 0 | | 346.4 | | | 15.2 | | |
| **No. of acids** | |  | | |  | | | 1 | | 0 | | 6 | | | 1 | | |
| **Terpenoinds** | | | | | | | | | | | | | | | | | |
| Myrcene | | 123-35-3 | | | C10H16 | | | ND | | 25.6±2.94 | | ND | | | ND | | |
| Limonene | | 138-86-3 | | | C10H16 | | | ND | | 1.6±0.22 | | ND | | | ND | | |
| β-limonene | | 5989-54-8 | | | C10H16 | | | ND | | ND | | 15.2±1.60 | | | ND | | |
| (E, E)-α-Farnesene | | 502-61-4 | | | C15H24 | | | ND | | 8.0±1.49b | | 32.8±4.27a | | | ND | | |
| (E)-Nerolidol | | 40716-66-3 | | | C15H26O | | | 124.8±13.75d | | 964.8±39.12b | | 1187.2±29.10a | | | 542.4±33.82c | | |
| Linalool | | 78-70-6 | | | C10H18O | | | 611.2±47.06b | | 1226.4±53.39a | | 440.8±16.06bc | | | 348.8±16.41d | | |
| **Total concentrations of terpenoids** | |  | | |  | | | 736 | | 2226.4 | | 1676.0 | | | 891.2 | | |
| **No. of terpenoids** | |  | | |  | | | 2 | | 5 | | 4 | | | 2 | | |
| **Furans** | | | | | | | | | | | | | | | | | |
| Furan, 2-pentyl- | | 3777-69-3 | | C9H14O | | | ND | | ND | | | | 37.6±3.77a | | | | 19.2±2.05b |
| 2,5-Dimethyl-4-hydroxy-  3(2H)-furanone | | 3658-77-3 | | C6H8O3 | | | ND | | ND | | | | 36.0±3.20a | | | | 27.2±1.99b |
| Mesifurane | | 4077-47-8 | | C7H10O3 | | | ND | | 126.4±12.48 | | | | ND | | | | ND |
| **Total concentrations of** | |  | |  | | | 0 | | 126.4 | | | | 73.6 | | | | 46.4 |
| **Table S2.** Continued | |  | |  | | |  | |  | | | |  | | | |  |
| **furans** | |  | |  | | |  | |  | | | |  | | | |  |
| **No. of furans** |  | | |  | | | 0 | | 1 | | | | 2 | | | | 2 |
| **Lactones** |  | | |  | | |  | |  | | | |  | | | |  |
| γ-Decalactone | 706-14-9 | | | C10H18O2 | | | ND | | ND | | | | ND | | | | 61.6±7.05 |
| **Total concentrations of lactones** |  | | |  | | | 0 | | 0 | | | | 0 | | | | 61.6 |
| **No. of lactones** |  | | |  | | | 0 | | 0 | | | | 0 | | | | 1 |
| Letters are comparisons of cultivars within a cropping system. Means with the same letter are not significantly different (*P* = 0.05) by least significant difference ( LSD ) test.  N D, not detected. | | | | | | | | | | | | | | | | | |
