# Supplementary material for: The flavor and nutritional characteristic of four strawberry varieties cultured in soilless system
Source: Food Sci Nutr. 2016 Mar 10;4(6):858–68. doi: 10.1002/fsn3.346 (PMC5090650; doi:10.1002/fsn3.346)
Supplement: Supplementary file 5 — Table S3. Esters special of each variety in a soilless growing system. [file FSN3-4-858-s005.doc]

| Variety | Numbers | Esters special for each variety |
| --- | --- | --- |
| Benihoppe | 2 | Methyl valerate; 4-Octenoic acid, methyl ester |
| Tochiotome | 4 | Octyl acetate; linalyl acetate; methyl 2-hexenoate and 2-Heptanol, acetate |
| Sachinoka | 9 | Methyl 2-methylbutyrate; hexyl formate; methyl cinnamate; linalool, formate; 4-Hexen-1-ol, acetate; 2-heptadecanol, acetate; butanoic acid, 1-methyloctyl ester and hexanoic acid, 3-tridecyl ester |
| Guimeiren | 16 | ethyl valerate; hexyl 3-methylbutanoate; benzyl butyrate; ethyl caprylate; ethyl caprate; nonyl isovalerate; ethyl benzoate; methyl anthranilate; methyl p-tert-butylphenylacetate; linalyl butyrate; Octanoic acid, 3-hydroxy-, methyl ester; (Z)-pent-2-enyl butyrate; hexanethioic acid, S-propyl ester; 2-ethylhexyl hexanoate; 2-methylbutyl hexanoate; and propanoic acid, 2-methyl-, nonyl ester |

**Table S3-Esters special of each variety in a soilless growing system**
