# Supplementary material for: The flavor and nutritional characteristic of four strawberry varieties cultured in soilless system
Source: Food Sci Nutr. 2016 Mar 10;4(6):858–68. doi: 10.1002/fsn3.346 (PMC5090650; doi:10.1002/fsn3.346)
Supplement: Supplementary file 6 — Table S4. Principal components value of quality markers of strawberry. [file FSN3-4-858-s006.doc]

**Table S4**-Principal components value of quality markers of strawberry

| Variety | Component 1 | Component 2 | Synthetic component |
| --- | --- | --- | --- |
| Benihoppe | 28.59627 | 7.1001 | 14.7236 |
| Tochiotome | 40.2572 | -3.27597 | 18.9978 |
| Sachinoka | 59.2669 | 5.8818 | 32.2043 |
| Guimeiren | 50.8358 | -0.26923 | 24.1059 |
